# Special Issue: “*Advances in Sources, Extraction, Encapsulation, Bioavailability, and Health Benefits of Functional Food Ingredientsts*”

**DOI:** 10.3390/foods14050759

**Published:** 2025-02-23

**Authors:** Rossella Vadalà, Rosaria Costa, Nicola Cicero

**Affiliations:** 1 Department of Biomedical Dental Morphological and Functional Imaging Sciences, University of Messina, 98168 Messina, Italy; costar@unime.it (R.C.); ncicero@unime.it (N.C.); 2 Science4life S.r.l. Start Up, 98168 Messina, Italy

The Special Issue “*Advances in Sources, Extraction, Encapsulation, Bioavailability, and Health Benefits of Functional Food Ingredients*” reaches its conclusion, having successfully compiled a collection of nine articles. These contributions represent a valuable body of knowledge and provide significant insights into the rapidly evolving field of functional foods. This research area integrates innovative methods for sourcing, processing, and evaluating bioactive compounds, reflecting the dynamic and interdisciplinary nature of functional food science. The selected articles center on advancements in sustainable food production, innovations in health-promoting food, and technological breakthroughs. These works collectively showcase the remarkable progress being made in understanding and applying functional food ingredients to improve human health. As editors, we extend our sincere gratitude to the authors for their meaningful contributions, to the reviewers for their thorough evaluations, and to the journal’s editorial team for their steadfast support. It is our hope that this collection will inspire further research efforts and collaborations, ultimately advancing the science of functional foods and enhancing their applications in promoting better health outcomes. The field of functional foods is advancing at an unprecedented rate, fueled by increasing consumer awareness of the connection between diet and health, as well as by innovations in food science [1]. Research is focused on enhancing the bioavailability, stability, and efficacy of bioactive compounds through innovative encapsulation methods and clinical validation [2]. It highlights sustainable extraction techniques using underutilized resources and novel strategies to overcome bioavailability barriers. These studies collectively demonstrate the potential of functional food ingredients to prevent and manage chronic diseases, underscoring their role in promoting long-term health and well-being. Key topics include the positive impact of natural ingredients like sprouts on gut microbiota [3], sulforaphene-loaded nanoparticles for improved anticancer properties [4], advanced methods to preserve nutrients like β-carotene during food processing [5], and the exploration of novel, sustainable protein sources to meet global demands for eco-friendly food solutions [6]. While these advancements mark significant strides forward, challenges remain. Standardizing bioactivity assessments, scaling laboratory findings for industrial applications, and understanding the complex interactions of bioactive compounds within diverse food matrices are areas that require continued focus. Future priorities for research should include enhancing sustainable sourcing and processing methods, leveraging personalized nutrition informed by nutrigenomics, conducting robust clinical trials for validation, and deepening our understanding of food matrix interactions to optimize functional food designs [7].

Rossella Vadalà

Rosaria Costa

Nicola Cicero

*Guest editors*
